# TUG1 promotes the expression of IFITM3 in hepatocellular carcinoma by competitively binding to miR-29a

**DOI:** 10.7150/jca.57477

**Published:** 2021-10-02

**Authors:** Weiwei Liu, Qian Feng, Wenjun Liao, Enliang Li, Linquan Wu

**Affiliations:** Department of Hepatobiliary Surgery, the Second Affiliated Hospital of Nanchang University, 1 Mindle Road, Nanchang Jiangxi 330006, P.R. China.

**Keywords:** TUG1, miR-29a, IFITM3, HCC, biological function

## Abstract

**Purpose:** Numerous studies have demonstrated the important relationship of TUG1 with tumorigenesis. The present study investigated the role of TUG1 and its downstream genes miR-29a and IFITM3 in the occurrence and development of hepatocellular carcinoma (HCC). We found that both TUG1 and IFITM3 genes are highly expressed in HCC, whereas the expression of miR-29a is low in HCC. Downregulation of TUG1 reduces cell invasion, metastasis, and cell proliferation ability and promotes cell apoptosis. Simultaneous downregulation of miR-29a reverses this effect. Moreover, IFITM3, as the target gene of miR-29a, is positively regulated by TUG1. However, the adjustment relationship between these three components is still unknown and thus warrants further investigation. The objective of this study was to investigate the regulatory relationship between TUG1, miR-29a, and IFITM3 in human liver cancer.

**Patients and methods:** The expression of TUG1 and miR-29a in tumor tissues and adjacent non-tumor tissues of 65 patients with HCC was detected by real-time quantitative polymerase chain reaction (RT-qPCR). The migration and invasion of liver cancer cells were studied by the wound healing assay and the Transwell method, respectively. The apoptosis rate of HCC cells was detected by flow cytometry, and the proliferation rate of hepatoma cells was detected by the 5-ethynyl-2'-deoxyuridine (EdU) method. Immunofluorescence was used to detect the expression of TUG1 and IFITM3 in HCC-LM3 and HL-7702 cell lines. The relationship between TUG1 and miR-29a was detected using a double luciferase reporter assay and fluorescence *in situ* hybridization (FISH). Tumors were established *in vivo* by subcutaneous injection of HCC cells into nude mice and injection of these cells into the tail vein. Western blotting was used to quantify the biomarkers.

**Results:** The expression of TUG1 increased significantly in tumor tissues and HCC cells. Moreover, the expression of miR-29a in liver cancer tissues was significantly lower than that in normal human liver tissues. The expression of TUG1 in liver cancer tissue was negatively correlated with miR-29a. Knockdown of TUG1 weakened the invasion, migration, and proliferation of HCC cells, and enhanced their apoptosis. A simultaneous knockdown of miR-29a enhanced cell invasion, metastasis, and cell proliferation, whereas the apoptosis ability decreased. As a target gene of miR-29a, IFITM3 is not only negatively regulated by miR-29a, but also positively regulated by TUG1. Therefore, TUG1 regulates IFITM3 in HCC cells by competitively binding to miR-29a, thus affecting cell invasion, migration, proliferation, and apoptosis.

**Conclusion:** As a CeRNA, TUG1 competitively binds to miR-29a to regulate IFITM3 and promote the development of liver cancer. Downregulation of TUG1 can significantly inhibit the migration, invasion, and proliferation of liver cancer cells. Based on these results, we conclude that TUG1 could serve as a key gene to improve the prognosis of patients with HCC.

## Introduction

According to the latest statistics, liver cancer is the sixth most common cancer worldwide, and it is also the fourth leading cause of cancer death 1. In China, several individuals are affected by liver cancer. The most common liver cancer is hepatocellular carcinoma (HCC), which is reported to account for 75 to 85% of primary liver cancers 2. Liver cancer is a fatal disease. The primary treatment strategy for liver cancer consists of surgery and liver transplantation. However, most liver cancers are diagnosed at a later stage as advanced HCC, following the ideal time for treatment 3. This leads to unsatisfactory clinical efficacy and prognosis after treatment 4, 5. Therefore, it is essential to elucidate the molecular mechanisms of liver cancer progression, early diagnose liver cancer, and find HCC biomarkers and therapeutic targets.

Taurine upregulated gene 1 (TUG1) is a crucial gene whose function in retinal development was first proposed in 2005 6. Subsequently, lncRNAs were shown to be involved in carcinogenesis by altering chromatin structure and acting as a small RNA sponge, associated with the expression of multiple cancer-related pathways 7, 8. Although most studies have revealed the carcinogenic effects of this lncRNA, it has also been reported that compared with non-cancer samples, TUG1 is downregulated in non-small cell lung cancer samples 9-11. In triple-negative breast cancer samples, the expression of lncRNA was reduced. Furthermore, its expression in HER2 enrichment and basal-like subtypes is higher than that in luminal subtypes 12. The expression and function of TUG1 are different in tumors. Furthermore, its role in epithelial-mesenchymal transition and activation of the Wnt/β-catenin pathway has been explored in human cancers 10, 13. Although TUG1 has been shown to be a crucial factor in the progression of multiple tumors, it is unclear how its downstream target genes affect HCC progression; thus, its mechanism of action in HCC is unclear.

Small molecule ribonucleic acid (microRNA) is an endogenous (approximately 22 nucleotides) small non-coding RNA family that can inhibit translational expression of proteins by direct binding to 3' untranslated region (3' UTR) messengers expressing the ribonucleic acid (mRNA) target gene silencing post-transcriptional regulation of gene expression 14-16. It has been reported that microRNAs are closely related to the apoptosis of nerve cells. The expression of miRNA-29a is significantly reduced in a variety of tumors 17, 18, indicating its involvement in a variety of tumor progression processes along with an important role in tumor growth, metastasis, apoptosis, and proliferation 19-21. As a member of the microRNA family, miR-29a is expressed in several tumors and exerts a tumor suppressor effect 22-24. However, its mechanism of action in liver cancer remains elusive and thus is worthy of further study.

The interferon-inducible transmembrane protein 3 (IFITM3, also known as 18u) gene (together with IFITM1 and IFITM2) belongs to the IFITM gene family that is clustered on chromosome 11 and is also considered to be an antiviral gene 25, 26. Recently, an increasing number of studies have found that the expression of IFITM3 is associated with the prognosis of a variety of tumors, such as gastrointestinal tumors and glioma, and it is involved in cell migration, invasion, proliferation, apoptosis, and tumorigenesis 27-30. However, the mechanism of the clinical significance of abnormal expression of IFITM3 in HCC is still relatively unknown, and the upstream genes remain to be explored. The present study aimed to understand how TUG1 regulates the expression of IFITM3, thereby affecting the development of HCC.

Although the research on the treatment of liver cancer is continuing, there has been no breakthrough in the treatment of advanced liver cancer. Our previous studies have shown IFITM3 as a direct target of miR-29a and an important gene regulating the development of liver cancer. The purpose of the present study was to confirm the differential expression of TUG1 in HCC and to determine whether it regulates IFITM3 through miR-29a, thereby affecting tumor invasion, migration, proliferation, and apoptosis. The ultimate goal was to find biomarkers and therapeutic targets for early liver cancer.

## Methods

### Tissue specimens and microarray data

Sixty-five pairs of histologically confirmed liver cancer and adjacent cancer specimens were collected from the Second Affiliated Hospital of Nanchang University. The collected specimens required no chemotherapy, radiotherapy, or immunotherapy. The collected specimens were immediately stored in liquid nitrogen and tissue fixation solution. The study was performed according to the Helsinki Declaration of 1964 and all subsequent amendments. All patients received written informed consent from the Ethics Committee of the Second Affiliated Hospital of Nanchang University. Three hundred seventy-four cases of liver cancer and 50 cases of normal liver tissue were accessed via the StarBase database, and the expression of TUG1 was compared.

### Cell lines and cell culture

Normal liver cells (HL-7702) and two liver cancer cell lines (MHCC-97H and HCC-LM3) were selected. All cells were purchased from Shanghai Cell Research Institute (Shanghai, China). HCC-LM3 and MHCC-97H cell lines were cultured in high-glucose Dulbecco's modified Eagle's medium (DMEM) containing 10% fetal bovine serum (FBS), and HL-7702 cells were cultured in RPMI 1640 medium containing 10% FBS. The cells were maintained in an incubator at 37 °C, 5% CO_2_, and 95% humidity. Cells in the logarithmic stage were used.

### Cell transfection

TUG1 siRNA, miR-29a inhibitor, and negative control (NC) were purchased from Guangzhou RiboBio Biotechnology (Guangzhou). IFITM3 siRNA and NC were purchased from Shanghai Gene Pharmaceutical. MHCC-97H and HCC-LM3 cells were divided into the NC and treatment groups. The purchased interference fragments were first subjected to qRT-PCR to verify their effectiveness (Figure S2). The interference fragment and inhibitor for each gene were transfected into cells using the Lipofectamine 3000 kit (Invitrogen; Thermo Fisher Scientific, Inc., Waltham, MA, USA). The TUG1 siRNA sequence was as follows: TUG1-s1 sense: 5'-GTTGACCTTGCTGTGAGAA-3' and antisense: 5'-AACCTGGGAACCTTGGATTG-3'; TUG1-s2 sense: 5'-GCACCTGGAACCTCATCTA-3' and antisense: 5'-CATCACTGGCATATCTGCCT-3'; TUG1-s3 sense: 5'- GCCTCTATTCCTGTATGTA-3' and antisense: 5'- ATCTAGGAGTCTGTATACTG-3'. The IFITM3 siRNA sequence was as follows: IFITM3-s1 sense, 5'-CCA UUC UGC UCA UCG UCA UTT-3' and antisense, 5'-AUG ACGAUGAGCAGAAUGGTT-3'; IFTM3‑s2 sense, 5'‑GCUGAUCUU CCAGGCCUAUTT‑3' and antisense, 5'‑AUAGGCCUGGAA GAUCAGCTT‑3'.

### Quantitative reverse transcription-polymerase chain reaction (qRT-PCR)

Total RNA was extracted with TRIzol reagent, Remove genomic DNA and subsequently reverse transcribed into cDNA using a reverse transcription kit (Takara; Tokyo, Japan). Next, quantitative reverse transcriptase-polymerase chain reaction (qRT-PCR) was run using a PrimeScript RT kit (Takara). miRNA reverse transcription was carried out using MiR-XTM miRNA First-Strand Synthesis kit (Takara). PCR was performed using SYBR®Premix Ex Taq™ kit (Takara). The relative expression of each gene was normalized against the expression of housekeeping genes and calculated using the 2^-ΔCt^ method. The RiboBio-designed primers were against TUG1, IFITM3, GAPDH, miR-29a, and U6. Please see additional materials for primer sequence.

### Western blot

All proteins were extracted with radioimmunoprecipitation assay (RIPA) buffer and a protease inhibitor in a 100:1 ratio. Samples were separated by polyacrylamide gel electrophoresis (SDS-PAGE) and transferred into PVDC membrane (Millipore, Massachusetts, USA) for 2h. Then, the membranes were incubated with primary antibody at 4 °C overnight. Tris-HCl solution + Tween-20 (TBST) was used to wash the membranes three times for 10 mins. Subsequently, they were incubated with horseradish peroxidase-conjugated secondary antibody for 1 hr at room temperature. Finally, the blots were detected with enhanced chemiluminescence, and band intensities were measured with Quantity-One software (Bio-Rad, Hercules, CA, USA). The primary antibodies against IFITM3 (ab109429), Bax (ab32503), Bcl2 (ab32124), tubulin (ab15246), GAPDH (ab8245), N-cadherin (ab76011), and E-cadherin (ab40772) were purchased from Abcam. Western blotting was used to calculate the amount of expressed protein.

### Scratch test

Scratch experiments were performed to detect cell migration ability. First, the cells were seeded into a six-well plate. When the cells grew to 80 to 90% confluence, a 200-µL sterile tip was used to form a scratch in each well. Next, the separated cells were washed away with phosphate-buffered saline (PBS), and the width of the scratches at 0 h was observed under a microscope. The cells were cultured for 24 h in fresh medium. Next, the width of the scratches was measured twice to calculate the ratio of cell healing. The healing ratio of the scratches was calculated.

### Transwell migration and invasion assays

According to the manufacturer's instructions, the matrix glue was added, and 60 to 80 µL matrigel was added to the inner chamber. Next, the wells were placed in the incubator for 30 min. First, the cells were starved for 12 to 24 h, following which a cell suspension was prepared. Next, the cells were uniformly added to the inner chamber with or without matrigel. Subsequently, 500 µL of serum-containing medium (DMEM) was added to each well in the outer chamber and incubated further for 24 to 48 h in the incubator. Thereafter, the cells in the chamber were washed, the cells were fixed with formaldehyde, and the outer cells were stained with 0.1% crystal violet. Finally, am image was acquired with a microscope after the water had dried.

### Measurement of apoptosis via flow cytometry

The cells were cultured in suspension for 48 h and digested with trypsin without ethylenediaminetetraacetic acid (EDTA). Next, flow cytometry analysis was performed using the Annexin V-fluorescein isothiocyanate (FITC)/propidium iodide (PI) Apoptosis Detection Kit (BD Biosciences) according to the manufacturer's instructions. Data were collected on a BD FACS Canto system and analyzed using the Flow Jo software.

### Cell cycle assays

The cells were cultured in suspension for 48 h, digested with trypsin without EDTA, and flow cytometry analysis was performed using a cycle kit (BD Biosciences) according to the manufacturer's instructions.

### EdU assay

The cells were first seeded into a 96-well plate. Next, the cells were fixed and stained with 5-Ethynyl-2´-deoxyuridine (EdU) according to the manufacturer's instructions (RiboBio). Finally, the cells were observed and photographed under a fluorescence microscope. Blue represented all cells, red represented proliferating cells, and the EdU-positive rate was indicated by the ratio of red cells to blue cells.

### *In vivo* experiment

Adult male nude mice of six to eight weeks of age were purchased from Hunan SJA Experimental Animal Co., Ltd. (Hunan, China). Nude mice were injected with a phosphate solution containing 1 × 10^7^ cells. Tumor volume was measured with calipers every four days and using the formula: tumor volume = (shortest diameter 2 × longest diameter)/2. After four weeks of photographing, the mice were anesthetized, the tumors were collected and weighed, and the expression of IFITM3 in the subcutaneous tumors was detected by immunohistochemistry, qRT-PCR, and western blotting. To assess lung metastasis, a phosphate solution containing 1 × 10^6^ HCC-LM3 cells was injected into the tail vein of nude mice. Three weeks later, the mice were sacrificed after anesthesia, and nude mice lung tissues were stained using hematoxylin and eosin (H&E) and immunized.

### Hematoxylin & eosin and immunohistochemical staining

To prepare tissue samples for immunohistochemistry, tissue samples were fixed with a tissue fixation fluid, placed in a paraffin block, and cut into paraffin sections. Dewaxing was first performed with xylene, and subsequently, the tissue sections were dehydrated with gradient alcohol. Sections were stained with H&E to determine if their morphology changed and next rehydrated and microwaved in a sodium citrate buffer (10 mmol/L, pH 6.0) to restore the antigen. Sections were incubated with 0.3% hydrogen peroxide/PBS for 30 min and then blocked with serum. Subsequently, tissue samples were incubated with a 1:200 dilution of rabbit monoclonal anti-IFITM3 antibody (ab15592, Abcam, Cambridge, MA, USA) at 4 °C overnight. The samples were then washed thrice with PBS for 5 min each and incubated with secondary antibody at 37 °C for 30 min. Next, the sections were stained with diaminobenzidine (DAB) and hematoxylin dye; the excess dye was rinsed with running water and then rehydrated with gradient alcohol, and sealed with neutral resin. Finally, the tissue samples were observed under the microscope, and images were acquired.

### Dual-luciferase reporter assay

The dual-luciferase reporter assay (DR) is an effective means to study the involvement of transcription factors in gene regulation. The DNA fragment of the promoter is analyzed to verify the transactivation ability of the promoter-binding element and study transcription. The molecular mechanism of the factor in signal transduction can be observed as the miRNA acts primarily through the 3' UTR on the target gene. Moreover, the 3' UTR region of the target gene can be constructed behind the reporter gene luciferase by comparing or overexpressing the miRNA. Changes in the expression of the reporter gene (monitoring changes in the luciferase activity) can quantitatively reflect the inhibitory effect of miRNA on the target gene, combined with site-directed gene mutations and other methods to further determine the site of action of the miRNA and the target gene 3' UTR. Dual-luciferase reporter assays were used to detect the binding between TUG1 and mir-29a-3p. wt and mutation sequences provide the following core binding domain: aagcgggttttgaagctggtgcc; Binding domain after mutation: aaCcgCCAAAAgaaCcACCACcc.

### FISH assay

Fluorescence *in situ* hybridization (FISH) is a sensitive and accurate technique to detect multiple genes simultaneously. It can be used to determine the exact position of the target gene, the positional relationship between several genes, and the relationship between genes and telomeres. The relationship between genes and centromeres is essential for the construction of genetic maps. The first is the deformation of the probe and the specimen, which includes incubating the probe in a warm water bath at 75 °C for 5 minutes, immediately setting it to 0 °C for 5-10 minutes to denature the double-stranded DNA probe, fixing the specimen, dehydrating, and air-drying, etc. For processing, 10 mL of denatured or pre-annealed DNA probe was dropped on the denatured and dehydrated slide specimen for hybridization; then washed and decolored; then amplified the hybridization signal for observation; finally, slices were sealed and observed under a fluorescence microscope. The nucleus stained by DAPI appears blue under the excitation by ultraviolet light, and the positive expression refers to the corresponding fluorescein-labeled fluorescence. FAM (488) appears green on excitation, and Cy3 appears red. The cy5-labeled probe showed specificity to TUG1, whereas the farm-labeled probe showed specificity to miRNA. The nuclei were stained with 4',6-diamidino-2-phenylindole (DAPI). All procedures were conducted according to the instructions of FISH kit (GenePharma) The TUG1 probe used was as follows: 5'-DIG-AATCTACCTCCAGTGTTCCTGCCGCATCGTG-DIG-3'. The miR-29a-3p probe used was as follows: 5'-DIG-TAACCGATTTCAGATGGTGCTA-DIG-3'.

### Immunofluorescence assay

The antigen-antibody reaction is based on the combination of the antibody with some tracer to locate the antigenic substance in the tissue or cell. Immunofluorescence steps include cell fixation and permeation, blocking, and incubation with primary and secondary antibodies. Immunofluorescence was used to localize the expression of TUG1 and IFITM3 (intranuclear or extranuclear).

### Database access

The expressions of TUG1 and IFITM3 were searched from the TCGA database. The prognostic analysis of TUG1 was obtained using the online tool UALCAN database, and the expression of TUG1 and TNM staging were searched in the GEPIA database. The relationship graph of TUG1 and miR-29a expression is obtained in the Starbase database. Tangetscan (http://www.targetscan.org/) predicts whether there is a binding site between miR-29a and IFITM3.

### Statistical analysis

Statistical analysis of the data was performed using GraphPad Prism 7.0 and SPSS 22.0. The expression of TUG1 in HCC tissues and adjacent tissues was compared using the Wilcoxon paired test. The correlation between TUG1 and miR-29a expression was found to be statistically significant using Spearman's correlation analysis. Differences in the overall survival were assessed using the Log-rank (Mantel Cox) test. The t-test was used to analyze the difference in the expression of tumor tissues and paracancerous tissues. The chi-square test was used to compare the data from the two groups. A p-value of less than 0.05 was considered statistically significant.

## Results

### TUG1 is highly expressed in HCC and shows poor prognosis

**Figure 1:**
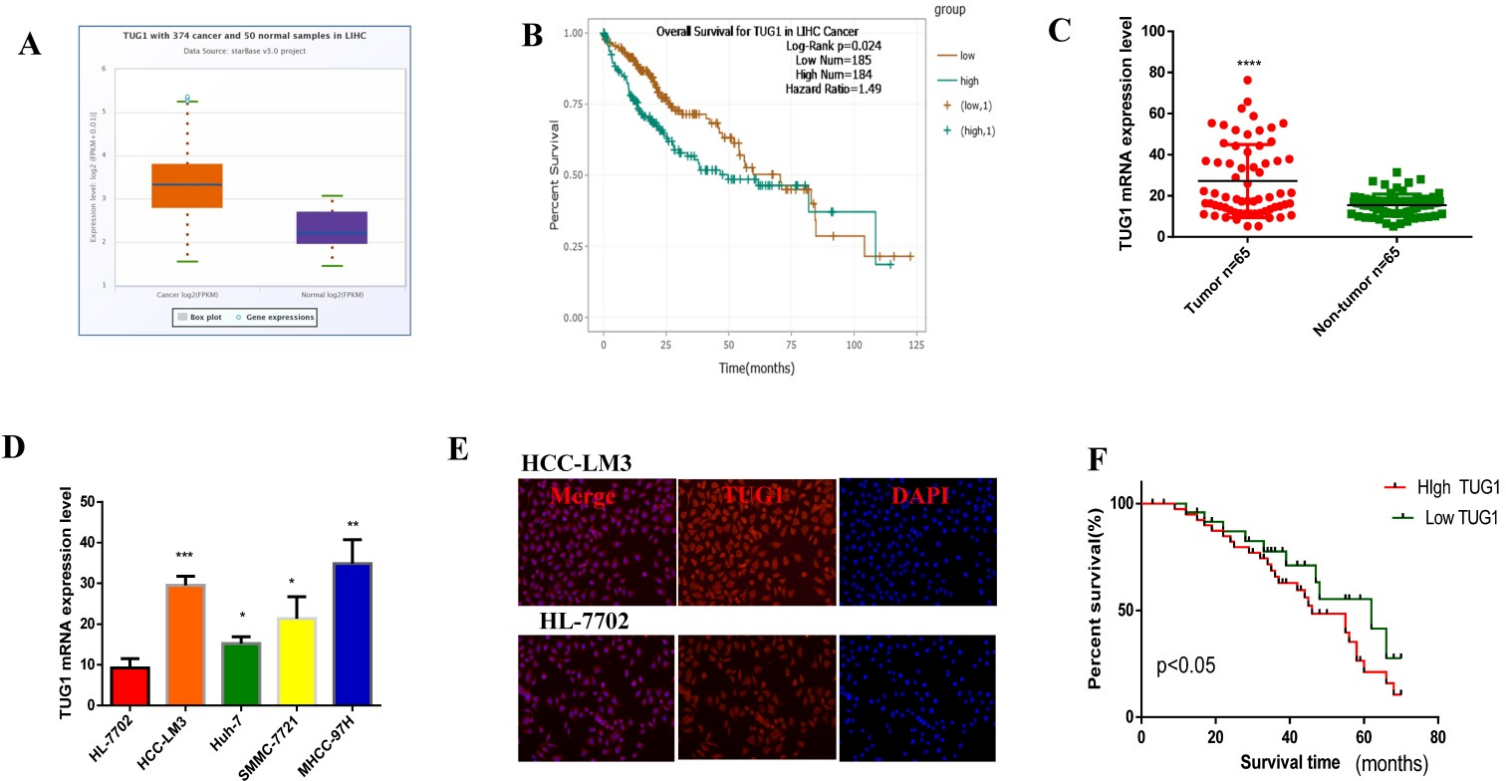
** TUG1 is highly expressed in HCC and shows poor prognosis. A, B.** The database shows that TUG1 is highly expressed in HCC and has a poor prognosis. **C.** qRT-PCR is used to detect the expression of TUG1 in liver cancer tissues and adjacent tissues. **D.** qRT-PCR is used to detect the expression of TUG1 in HCC cell lines and HL-7702. **E.** Immunofluorescence analysis of the expression of TUG1 in HCC-LM3 and HL-7702, the scale bar is 200 mm. F. The prognosis of patients with high TUG1 expression was significantly lower than that in patients with low TUG1 expression (p < 0.05). Formula of expression: 2^-ΔCt^. *p <0.05, **p <0.01, ***p <0.001, ****p <0.0001.

In order to investigate the expression of TUG1 in HCC, we first found the abnormal expression of TUG1 in LIHC in the database (Figure 1A), and its expression was also related to the poor prognosis of HCC Figure 1B). Furthermore, we found that the expression of TUG1 was closely related to the grade of the tumor from the database (Figure S1A). Then, we recorded the clinicopathological characteristics of 65 patients with HCC, including age, gender, tumor size, tumor node metastasis stage, tumor multifocality, venous invasion, HBsAg infection, alpha fetoprotein indicators, and cirrhosis. The results are shown in Table 1. We can conclude that the expression of TUG1 is significantly related to the tumor size, TNM stage, and tumor venous invasion (Table 1). We confirmed that TUG1 was up-regulated in HCC tissues and adjacent tissues by qRT-PCR (Figure 1C), and the same result was also confirmed in HCC cells (Figure 1D). We also found that the expression of TUG1 in HCC-LM3 cells was significantly higher than that in HL-7702 cells by immunofluorescence (Figure 1E). The results of qRT-PCR and prognostic follow-up indicated that the expression of TUG1 in HCC tissues was significantly higher than that in the adjacent tissues. The higher the level of TUG1 expression is, the worse the prognosis of patients would be (Figure 1F).

### Downregulation of TUG1 can weaken cell invasion and metastasis, proliferation, and enhance cell apoptosis

**Figure 2:**
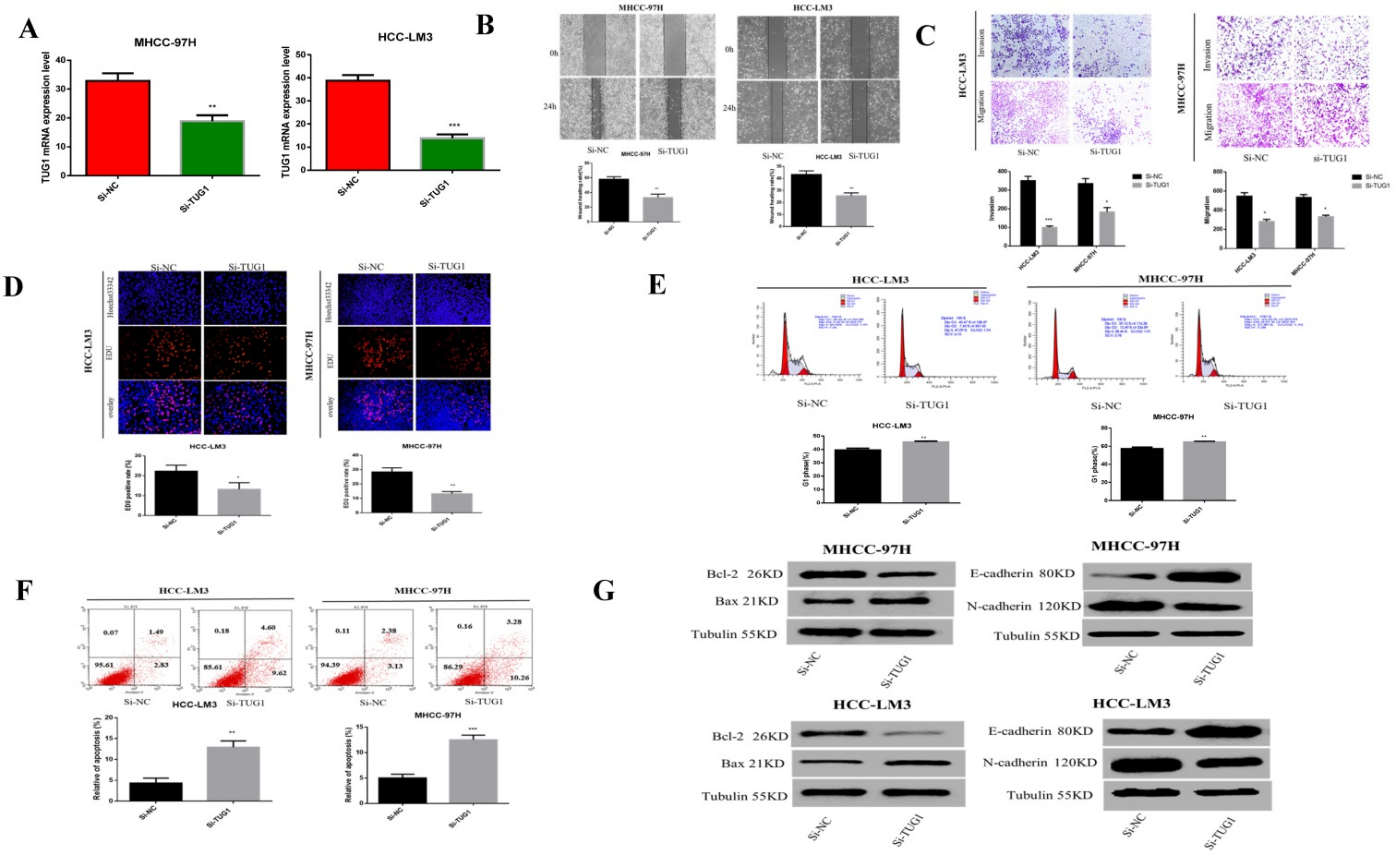
** Downregulation of TUG1 can reduce the malignancy of HCC cells. A.** The level of TUG1 mRNA after transfection of cells with si-TUG1 was detected using qRT-PCR. **B.** The effect of downregulating TUG1 on cell migration ability in HCC-LM3 and MHCC-97H cells was observed by wound healing experiments. **C.** The migration and invasion abilities of transfected si-TUG1 to HCC-LM3 and MHCC-97H cells were assessed by Transwell migration and matrix gel invasion assays. **D.** EdU experiments detected changes in the proliferation ability after the transfection of cells with si-TUG1. **E.** Changes in apoptotic rate after si-TUG1. **F.** Cyclic changes in HCC cells after si-TUG1. **G.** Changes in related proteins after transfection of si-TUG1.

Previous studies have reported that TUG1 is highly expressed in liver cancer and is related to the subsequent negative effects of HCC. Therefore, we speculate that TUG1 plays a crucial role in the carcinogenesis of HCC. Before the cell function experiments, we tested the down-regulation effect of TUG1 siRNA (Figure 2A). Scratch experiments showed that the downregulation of TUG1 reduced the ability of cell transfer (Figure 2B). Furthermore, the Transwell method showed that the downregulation of TUG1 reduced cell invasion and migration (Figure 2C). EdU assay proved that the downregulation of TUG1 reduced cell proliferation (Figure 2D). The cell cycle experiment showed that downregulating TUG1 increased the proportion of cells in G1 (Figure 2E). Flow cytometry experiments showed that TUG1 downregulation increased the apoptosis rate of HCC cells (Figure 2F). Furthermore, we assessed related proteins to further prove our findings (Figure 2G). The results showed that TUG1 is a consistent oncogene, and its downregulation reduces its malignancy. Therefore, we believe that the downregulation of TUG1 can inhibit HCC cell invasion, migration, and proliferation, and promote cell apoptosis.

### Relationship between TUG1 and miR-29a

**Figure 3:**
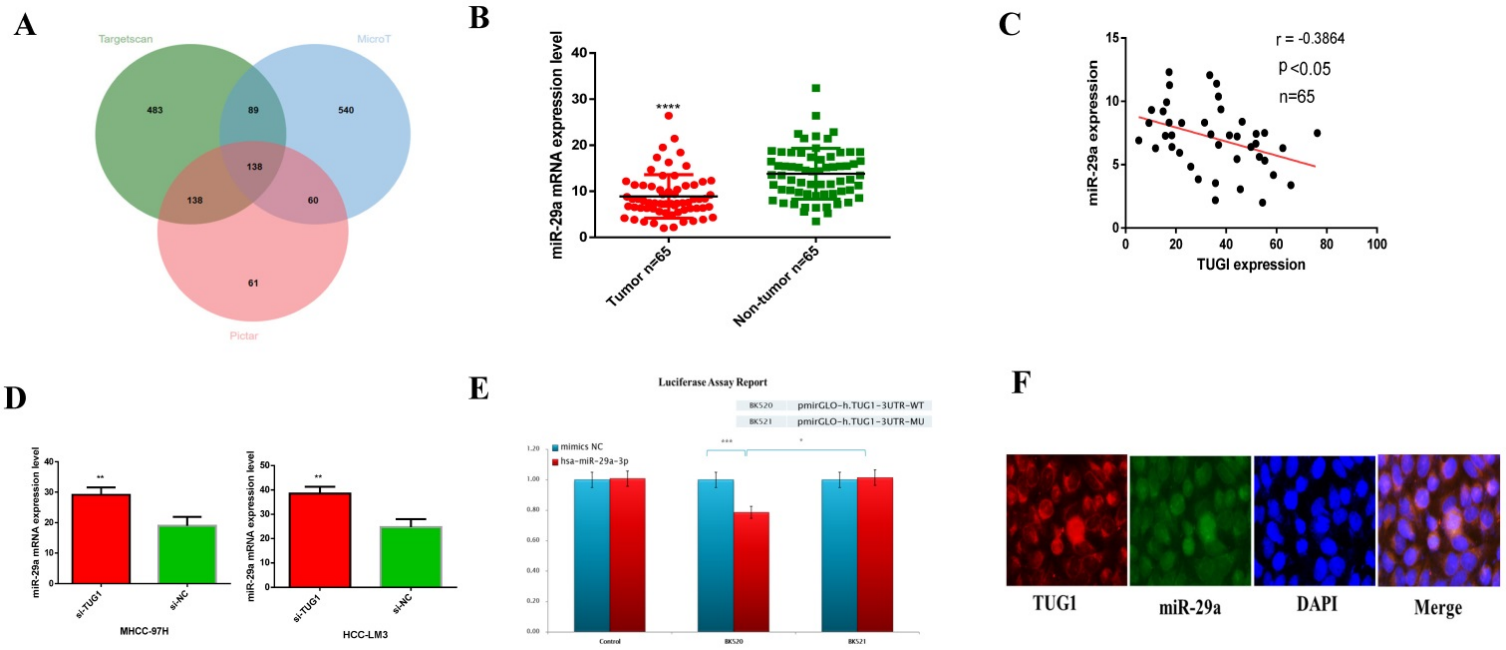
** The relationship between TUG1 and miR-29a. A.** The downstream target gene of TUG1 was predicted by Venn plot analysis using TargetScan, microT, and Pictar in three ellipses. The target gene was found at the intersection of the three databases. **B.** qRT-PCR was used to detect the expression of miR-29a in liver cancer tissues and adjacent tissues (n=65). **C.** TUG1 and miR-29a expression diagram (n=65). **D.** The expression of miR-29a after down-regulating TUG1. **E.** Dual luciferase reporter gene detection revealed that TUG1 was bound to miR-29a-3p. WT, wild‑type; MU, mutant type. **F.** The merged images showed that TUG1 and miR-29a are co-localized in HCC tissues using FISH, the scale bar is 200 mm.

We use DIANA tools (http://carolina.imis.athena-innovation.gr/) to analyze and find miRNAs that may be related to TUG1. I picked some of the results and plotted them into a table (Table S1), and found through correlation analysis TUG1 may have the closest relationship with miR-29a, so we chose miR-29a among related miRNAs.Through multiple data sorting, it was found that miR-29a is one of the downstream target genes of TUG1. Moreover, the Venn diagram of the downstream target genes of TUG1 was constructed (Figure 3A). Next, we checked the expression of miR-29a in liver cancer specimens by qRT-PCR (Figure 3B) and constructed a scatter plot with TUG1 expression (Figure 3C). The results showed that the expression of TUG1 was negatively correlated with the expression of miR-29a. This result is consistent with our findings from the database (Figure S1B). Next, we found that the expression of miR-29a can be increased by knocking down TUG1 (Figure 3D). We speculate that miR-29a is a downstream target gene of TUG1 and is negatively regulated by TUG1. In order to verify our conjecture, we made a dual luciferase report for both TUG1 and miR-29a. The report showed: **1)** After the action of hsa-miR-29a-3p, TUG1 3'UTR activity decreased by 21% (P<0.001), indicating that hsa-miR-29a-3p can act on the TUG1 3UTR region. **2)** After the TUG1 3'UTR is mutated, under the action of hsa-miR-29a-3p, the 3UTR activity is 23% higher than that of the wild type (P<0.05), indicating that the mutation site is very important for the binding of hsa-miR-29a-3p (Figure 3E). In order to ensure the accuracy of the experimental results, we also did a fluorescent probe *in situ* hybridization double labeling (double FISH) experiment to verify again, the results also showed that TUG1 and miR-29a-3p are mutually binding (Figure 3F). In summary, we determined that miR-29a is a direct target gene downstream of TUG1.

### miR-29a can reverse the invasion and metastasis of HCC cells by TUG1, promotion of proliferation, and inhibition of apoptosis

**Figure 4:**
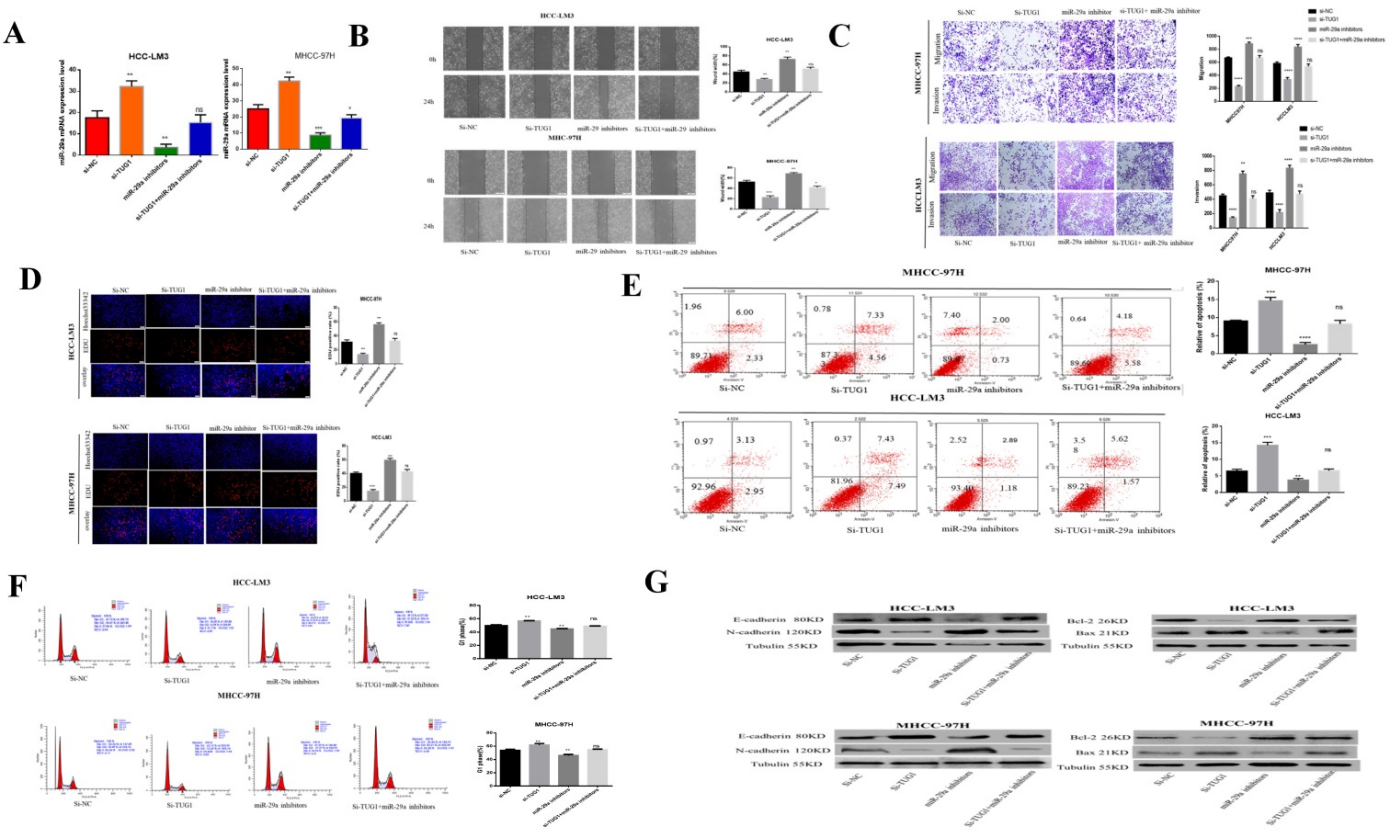
** miR-29a can reverse the invasion and metastasis of HCC cells by TUG1, the promotion of proliferation, and the inhibition of apoptosis. A.** qRT-PCR was used to detect the level of miR-29a mRNA after transfection with si-TUG1 and miR-29a inhibitors. **B.** The effects of TUG1 and miR-29a on cell migration ability were observed by wound healing experiments in HCC-LM3 and MHCC-97H cells. **C.** The migration and invasion abilities of HCC-LM3 and MHCC-97H cells transfected with si-TUG1 and miR-29a inhibitors were evaluated by Transwell migration and matrix gel invasion assays. **D.** EdU assay was used to detect changes in the proliferative capacity after the transfection of cells with si-TUG1 and miR-29a inhibitors. **E.** Changes in the apoptotic rate of si-TUG1 and miR-29a inhibitors. **F.** Changes in the cell cycle of si-TUG1 and miR-29a inhibitors. **G.** Changes in the expression of related proteins after transfection of cells with si-TUG1 and miR-29a inhibitors.

Our previous studies have determined that miR-29a is a downstream target gene of TUG1. The role played by miR-29a in regulating TUG1 is the focus of our next research. To study the interaction between TUG1 and miR-29a in liver cancer, we first transfected TUG1 siRNA and miR-29a inhibitor into HCC-LM3 and MHCC-97H cells and subsequently detected the expression of miR-29a mRNA. TUG1 could reverse regulate miR-29a (Figure 4A). The scratch test showed that the downregulation of TUG1 reduced the metastatic ability of cells, whereas the downregulation of miR-29a enhanced the metastatic ability of cells. Simultaneous downregulation of TUG1 and miR-29a had no significant difference in the metastatic cell ability as compared with the control group (Figure 4B). The Transwell assay proves that the downregulation of TUG1 reduced cell invasion and migration, whereas the downregulation of miR-29a exerted the reverse effect (Figure 4C). The EdU assay demonstrated that the downregulation of TUG1 reduced cell proliferation, whereas the downregulation of miR-29a enhanced cell proliferation (Figure 4D), downregulating TUG1 increased cell apoptosis and blocked cells in G1 phases, but these results can be reversed by miR-29a inhibitors (Figure 4E, 4F). We also checked related proteins to further validate our results (Figure 4G). Based on these results, we conclude that TUG1 is an oncogene in HCC, and the downregulation of miR-29a can enhance the malignancy of TUG1.

### miR-29a can negatively regulate the expression of IFITM3 in HCC

**Figure 5:**
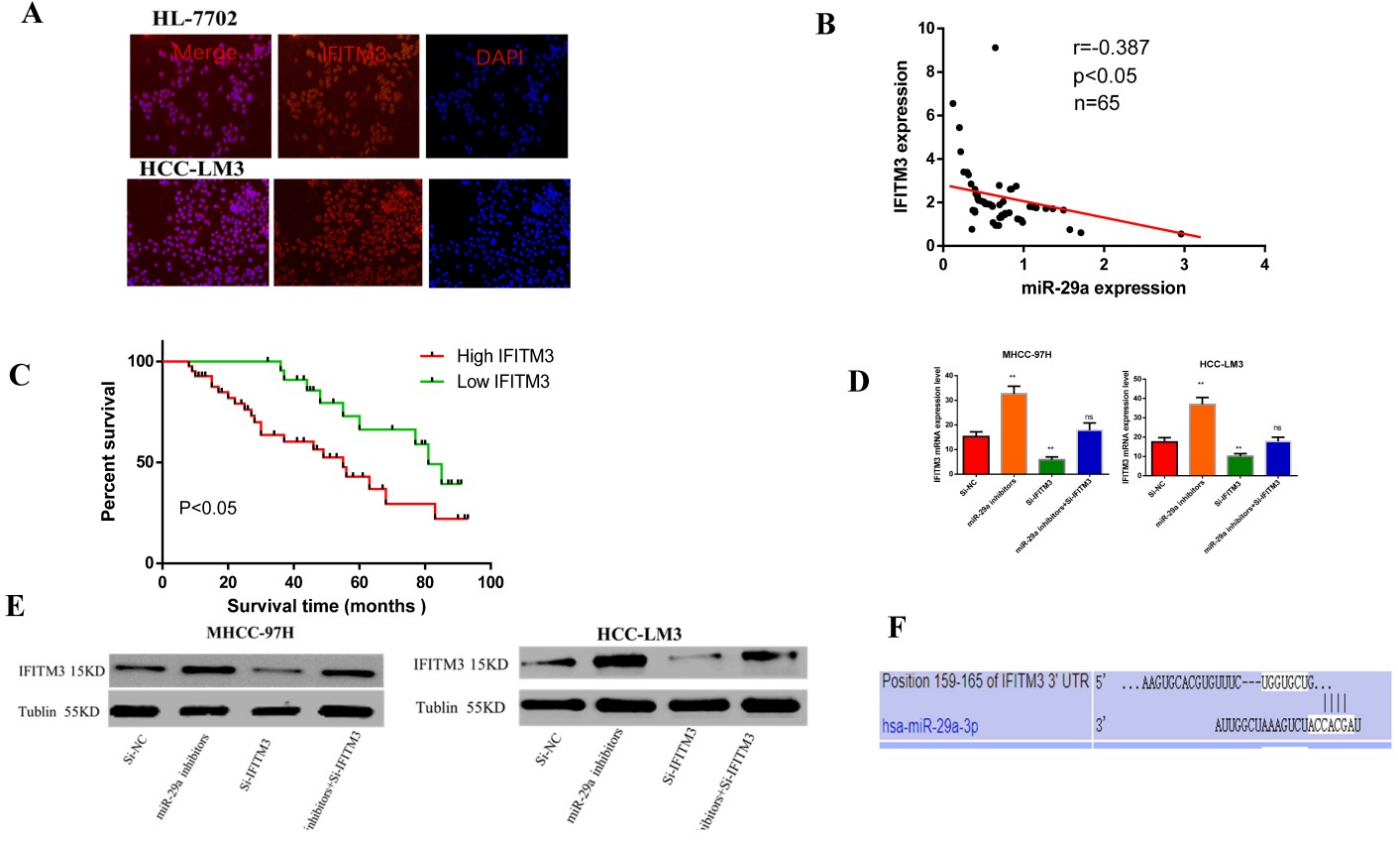
** Relationship between miR-29a and IFITM3. A.** qRT-PCR was used to detect the expression of miR-29a and IFITM3 in HCC. **B.** The changes of IFITM3 mRNA level after miR-29a inhibitors and IFITM3 siRNA transfection. **C.** The changes of IFITM3 protein levels after the siRNA transfection of miR-29a inhibitors and IFITM3 were used. **D.** Targetscan website was used to predict the relationship between miR-29a and IFITM3. **F.** Double Luciferase Report was used to analyze the relationship between miR-29a and IFITM3.

**Figure 6:**
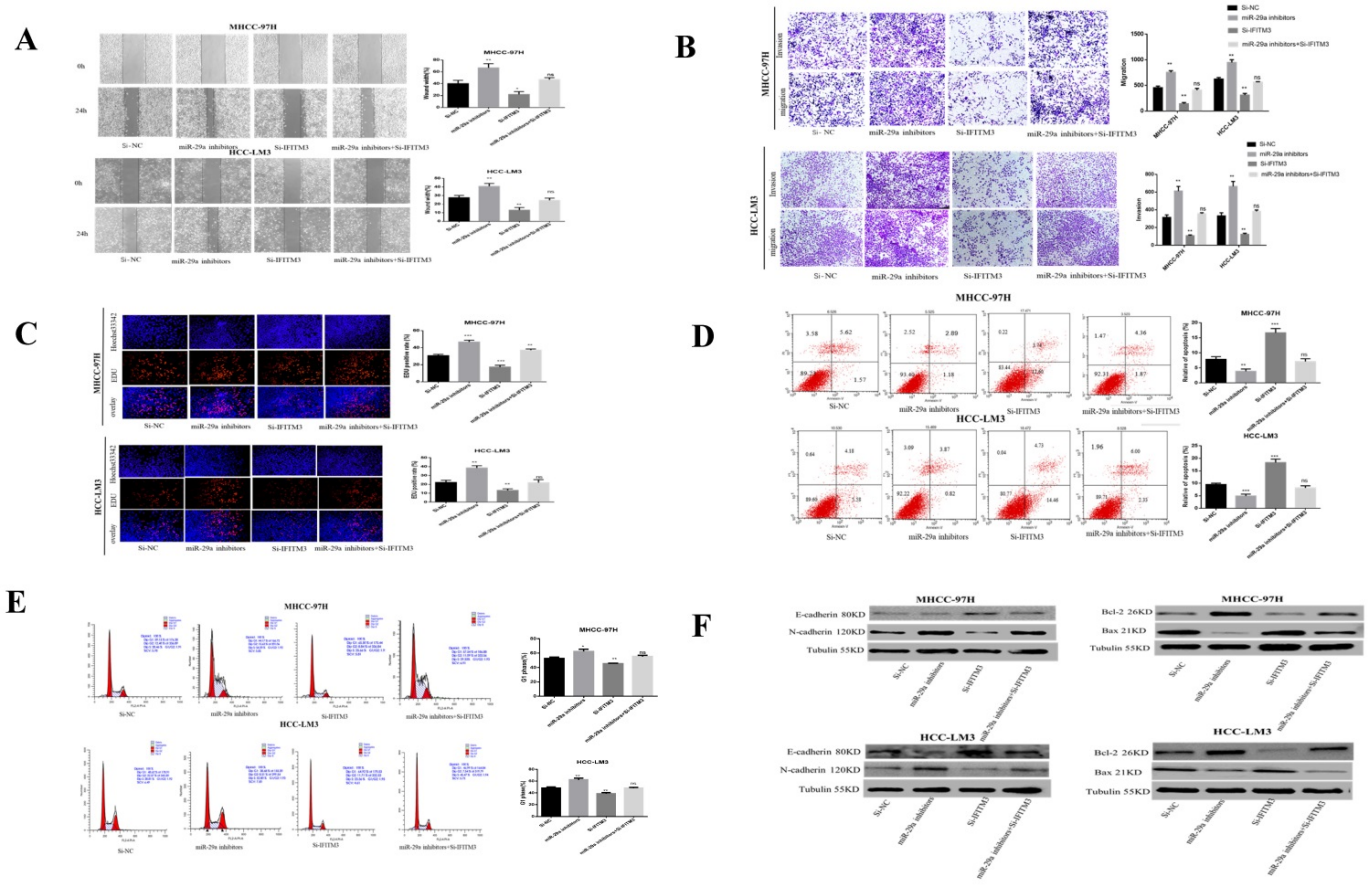
** miR-29a can negatively regulate the expression of IFITM3 in HCC. A.** The effect of transfection of miR-29a inhibitors and IFITM3 siRNA on HCC cells wound healing experiments. **B.** The migration and invasion abilities of transfected miR-29a inhibitors and IFITM3 siRNA in HCC-LM3 and MHCC-97H cells were evaluated by the Transwell migration and matrix gel invasion assay. **C.** EdU experiments were used to detect changes in the proliferative capacity after the transfection of cells with miR-29a inhibitors and IFITM3 siRNA. **D.** Changes in the apoptotic rate of miR-29a inhibitors and IFITM3 siRNA cells. **E.** Transfection of miR-29a inhibitors and IFITM3 siRNA cell cycle changes. **F.** Changes in the levels of related proteins after transfection of cells with miR-29a inhibitors and si-IFITM3.

Our previous study had found that IFITM3 promotes HCC progression, and might be a target gene of miR-29a 31 (PMID: 30272306). In order to study the interaction between miR-29a and IFITM3 in HCC, by immunofluorescence, we first detected that the expression of IFITM3 in HCC-LM3 was significantly higher than that in HL-7702(Figure 5A). Then we detected a negative correlation between the expression of miR-29a and IFITM3 in HCC tissues by qRT-PCR (Figure 5B) and we found that the expression of IFITM3 was related to TNM stage and venous invasion through clinicopathological and prognostic follow-up (Table 2), and the prognosis of patients with high expression of IFITM3 was significantly worse than that of patients with low expression of IFITM3 (Figure 5C). Then, we detected the changes of IFITM3 mRNA and protein levels after transfection of miR-29a inhibitors and si-IFITM3 inhibitors for 36 hours. We found that the mRNA and protein levels of IFITM3 increased after transfection with miR-29a inhibitor (Figure 5D, 5E). Therefore, we found that miR-29a can inhibit the expression of IFITM3. Next, we predicted that IFITM3 might be the downstream target gene of miR-29a on targetscan website (http://www.targetscan.org/) (Figure 5F). Next, we verified our hypothesis through functional experiments. Scratch test showed that the downregulation of miR-29a enhanced the metastatic ability of the two cell lines, whereas the downregulation of IFITM3 reduced the metastatic ability of the cells (Figure 6A). Transwell assay showed that downregulation of miR-29a enhanced cell invasion and migration ability, whereas the downregulation of IFITM3 exerted an opposite effect (Figure 6B). EdU test showed that downregulation of miR-29a enhanced cell proliferation, whereas the downregulation of IFITM3 reduced cell proliferation (Figure 6C). Flow cytometry showed that downregulation of miR-29a inhibited apoptosis, whereas the downregulation of IFITM3 promoted apoptosis. The downregulation of cell cycle assessment also showed the opposite result (Figure 6D, 6E). We further confirmed our observation by western blotting (Figure 6F). Therefore, in conclusion, we confirm that IFITM3 is negatively regulated by miR-29a as an oncogene in HCC, that is, miR-29a can inhibit the expression of IFITM3. Decrease the expression of miR-29a can reverse the expression of IFITM3 and promote the progress of tumor.

### TUG1 regulation of IFITM3 in HCC cells

**Figure 7:**
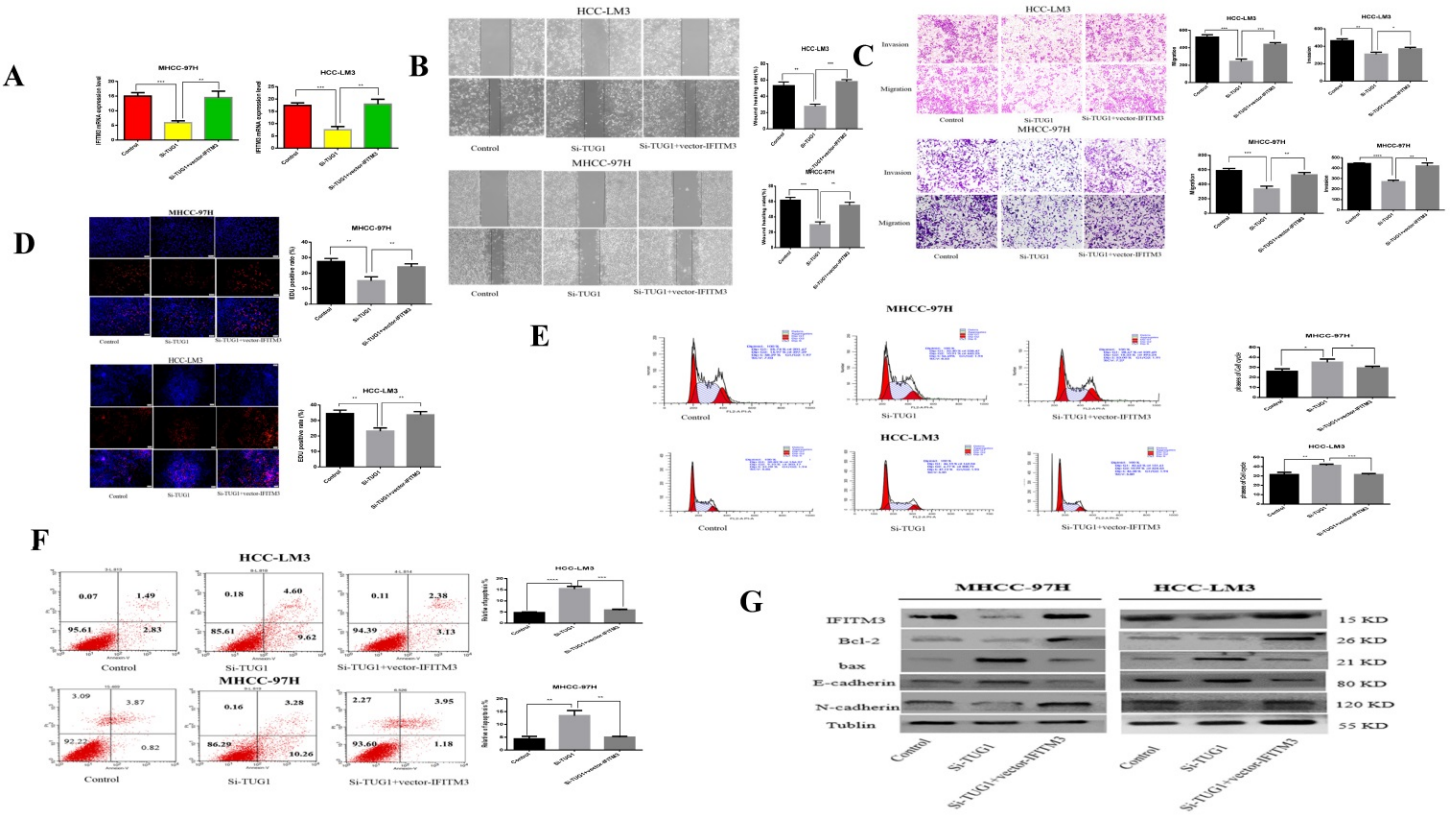
** TUG1 regulates IFITM3 expression to promote migration and invasion of HCC cell lines *in vitro*. A.** qRT-PCR detects the level of IFITM3 mRNA after transfection of si-TUG1 and IFITM3 Vector. **B.** The effect of down-regulation of TUG1 and up-regulation of IFITM3 on cell migration was observed through scratch experiments. **C.** The migration and invasion abilities of transfected si-TUG1 and IFITM3 Vector to HCC-LM3 and MHCC-97H cells were evaluated by the Transwell migration and matrix gel invasion assay. **D.** EdU experiments were used to detect changes in proliferative capacity after transfection of si-TUG1 and IFITM3 Vector. **E.** Transfection of si-TUG1 and IFITM3 Vector cell cycle changes.** F.** Changes in the apoptotic rate of si-TUG1 and IFITM3 Vector cells. **G.** Changes in related proteins after transfection of si-TUG1 and IFITM3 Vector. *p < 0.05, **p < 0.01, ***p < 0.001, ****p < 0.0001.

Our previous studies showed that miR-29a can reverse-regulate IFITM3. We speculate that TUG1 can positively adjust IFITM3. First, we found that IFITM3 is highly expressed in liver cancer tissues through the GEPIA2 database (Figure S1C). At the same time, we also verified this result by qRT-PCR (Figure S1D). Next, we made a diagram showing the relationship between TUG1 and IFITM3 expression using the results of qRT-PCR (Figure S1E). We found through qRT PCR that when TUG1 is down-regulated, IFITM3 will also decrease, but when IFITM3 is also up-regulated, the regulatory effect of TUG1 on IFITM3 will be weakened or even disappear (Figure 7A). Next, we validated our hypothesis through functional experiments. The scratch test showed that when TUG1 is downregulated and IFITM3 is upregulated, the metastatic ability of cells will be restored (Figure 7B). Transwell experiments show that up-regulation of IFITM3 and down-regulation of TUG1 in HCC cells have opposite effects on cell migration and invasion (Figure 7C). EdU analysis showed that when TUG1 was down-regulated and IFITM3 was up-regulated, it could increase the cell proliferation ability (Figure 7D). At the same time, cells that down-regulated TUG1 and up-regulated IFITM3 had a higher level of apoptosis and weaker proliferation ability than cells that only down-regulated TUG1 (Figure 7E, 7F). We also used western blots to further validate our results (Figure 7G). Therefore, we believe that TUG1 can positively regulate IFITM3.

### TUG1 acts as a ceRNA and competitively binds miR-29a to regulate IFITM3

**Figure 8:**
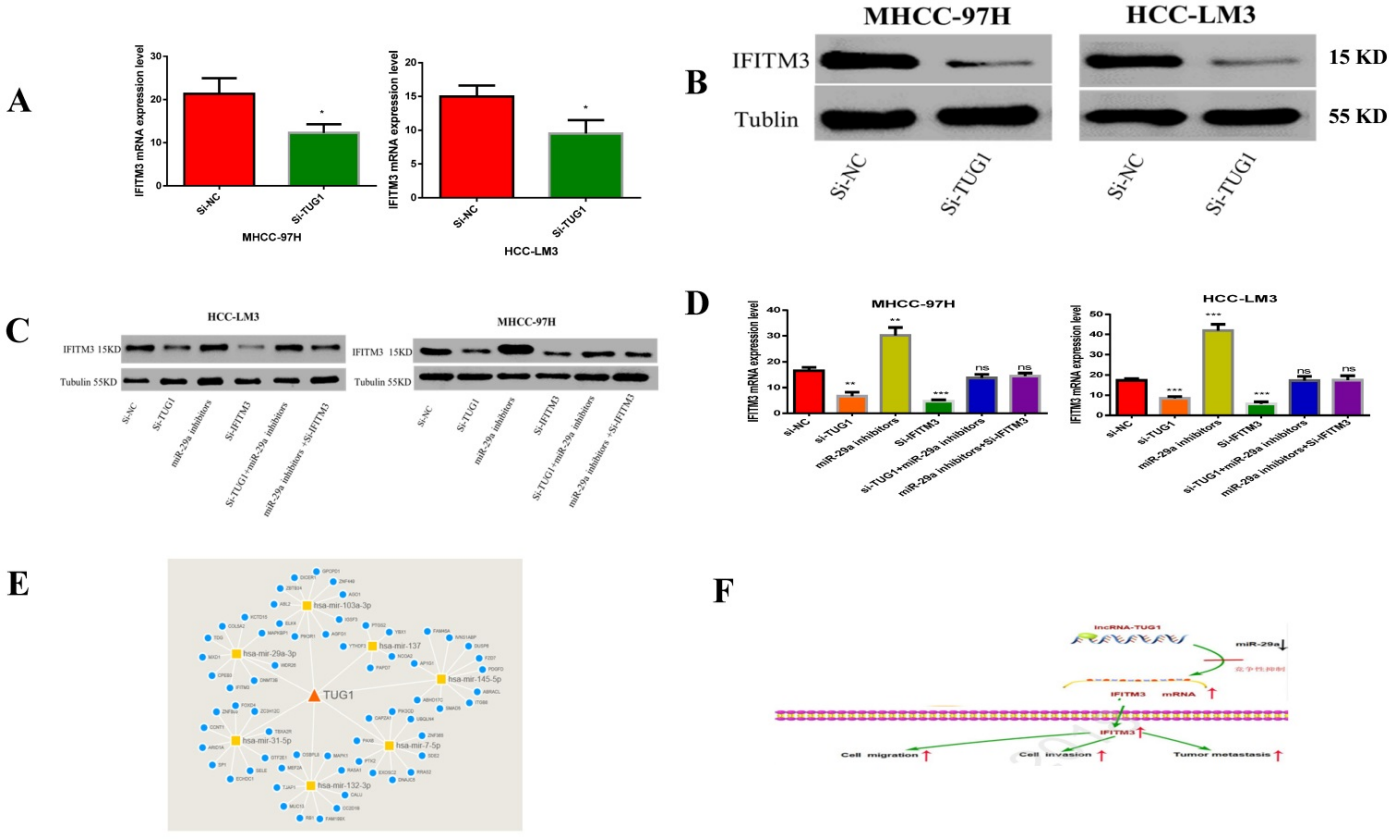
** TUG1 as a ceRNA competitively binds to miR-29a to regulate IFITM3. A, B.** T Downregulation of TUG1 decreased the levels of IFITM3 mRNA and protein. **C.** Transfection with TUG1 siRNA, miR-29a inhibitor, and IFITM3 siRNA, and changes in the expression of related proteins after transfection with TUG1 siRNA and miR-29a inhibitor, miR-29a inhibitor and IFITM3 siRNA. **D.** Transfection of cells with TUG1 siRNA, miR-29a inhibitor and IFITM3 siRNA, and simultaneous expression of IFITM3 mRNA after transfection of cells with TUG1 siRNA and miR-29a inhibitor, miR-29a inhibitor and IFITM3 siRNA. **E.** The results between the three through the collection of database information and the analysis of bioinformatics. **F.** The experimental simulation diagram.

In order to study the relationship between these three factors, we first determined that TUG1 could negatively regulate miR-29a (Figure 3 and 4), and subsequently determined the relationship between miR-29a and IFITM3 (Figure 5 and 6). More importantly, downregulating the expression of TUG1 reduced the expression of IFITM3 (Figure 8A,8B). Subsequently, we divided the experimental components into six groups, namely transfected si-NC, si-TUG1, miR-29a inhibitor, si-IFITM3, si-TUG1 + miR-29a inhibitor, and miR-29a inhibitor + si-IFITM3. After 36 h, the mRNA and protein levels of IFITM3 were checked. We found that the downregulation of TUG1 increased the expression of miR-29a but decreased the expression of IFITM3. Downregulating miR-29a increased the expression of IFITM3. Moreover, a simultaneous downregulation of TUG1 and miR-29a and a simultaneous downregulation of miR-29a and IFITM3, did not significantly change the levels of IFITM3 mRNA and protein as compared with the control group (Figure 8C, 8D). In addition, we found that TUG1, miR-29a and IFITM3 are related to each other through the collection of database information and bioinformatics analysis (Figure 8E). Through the above experiments, we confirmed that TUG1 can directly inhibit the expression of miR-29a, miR-29a can also inhibit the expression of IFITM3, and TUG1 can promote the expression of IFITM3. Combined with the analysis of clinical data, it is found that TUG1 and IFITM3 are both highly expressed in HCC tissues, and their expressions are positively correlated, while the expression of miR-29a in HCC is negatively correlated with both TUG1 and IFITM3. Based on these results, we infer that TUG1 promotes the expression of IFITM3 by inhibiting the expression of miR-29a. Therefore, we conclude that TUG1, as a ceRNA, competitively binds miR-29a to regulate IFITM3, thereby affecting the occurrence and development of HCC (Figure 8F).

### *In vivo* experiments

**Figure 9:**
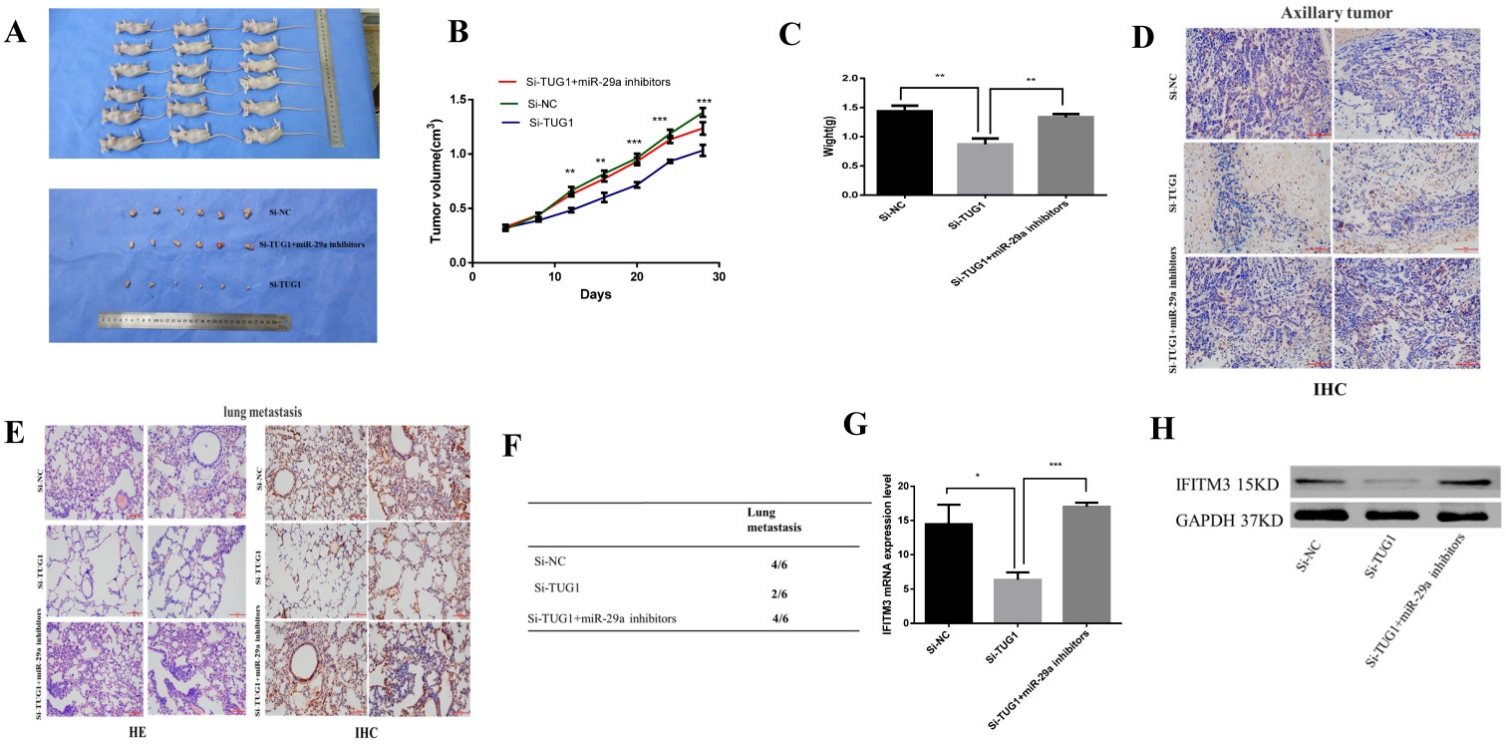
** TUG1 regulates miR-29a to promote tumorigenesis *in vivo*. A.** Nude mice were injected with HCC-LM3 stable cells. The tumor was dissected and photographed after five weeks.** B.** Changes in the tumor volume of mice were observed every four days. **C.** Mean tumor weight at the end in each group of experiments (Day 28). Data represent the mean, standard deviation (SD; n = 6), *p < 0.05. **D.** Immunohistochemistry was used to detect the expression of subcutaneous tumor IFITM3. **E, F.** Lung metastasis-related detection. H&E staining of lung tissue for histological analysis. Immunohistochemistry was used to detect the expression of IFITM3 in lung tissue. **G, H.** Mouse tumors were tested by qRT-PCR and western blotting to detect the expression of IFITM3.

To investigate the effects of TUG1 and miR-29a *in vivo*, we subcutaneously injected TUG1 knockdown cells, miR-29a knockout cells, or control cells into nude mice, and evaluated the tumor growth. The tumor growth rate of nude mice in the TUG1 downregulated group was significantly slower than that in the control group and the nude mice group with simultaneous downregulation of TUG1 and miR-29a (Figure 9A). The final size and weight of the tumor were also smaller than those in the case of the other two groups (Figure 9B, 9C). Next, we assessed the relationship between TUG1 and miR-29a *in vivo*. The immunohistochemical results showed that knocking down TUG1 significantly reduced the average area of IFITM3 immune-positive tumors (Figure 9D). The lung metastasis test showed that the degree of tumor metastasis and the average area of IFITM3 immune-positive lung tissue were significantly reduced after knocking down TUG1, whereas simultaneous knocking down TUG1 and miR-29a resulted in findings similar to those obtained in the control group (Figure 9E, 9F). In addition, by examining tumor tissue using qRT-PCR and western blotting (Figure 9G, 9H), we found that the expression of IFITM3 in tumor tissues of mice with TUG1 knockdown was significantly lower than that in the other two groups. These results indicate that TUG1 could influence miR-29a-mediated regulation of IFITM3 to promote tumor development *in vivo*.

## Discussion

The present study is the first to propose that TUG1 plays a crucial role in HCC. It acts as an oncogene and competitively binds to miR-29a as a ceRNA to regulate IFITM3, which in turn affects the development and progression of liver cancer.

Recently, with the development of bioinformatics and the development of high-throughput sequencing, long-chain non-coding RNAs have received increased attention. LncRNA is an RNA that is more than 200 nucleotides in length and lacks the ability to encode a protein 9, 32, 33. TUG1 is a newly found cancer-related lncRNA, which is abnormally expressed in various types of cancers, and functions as an oncogene or tumor suppressor gene. The biological functions of TUG1 in different tumors vary widely. TUG1, as an lncRNA, plays an important role in several tumors; however, its specific mechanism of action in liver cancer has not been studied. The results of qRT-PCR showed that the expression of TUG1 in liver cancer tissues and cells was significantly higher than that in adjacent tissues and normal hepatocytes. We found that the expression of TUG1 in liver cancer cells (HCC-LM3) was significantly higher than that in the normal cells and hepatocytes (HL-77O2), which is consistent with the previously reported results. Next, we performed biological function experiments by transfecting TUG1 siRNA and found that downregulation of TUG1 reduced the invasion, metastasis, and proliferation, and enhanced the apoptosis of HCC cells. In our prognostic follow-up analysis, the survival time of patients with high expression of TUG1 was significantly shorter than that in patients with low expression. It can be seen that TUG1 plays a crucial role as an oncogene in liver cancer. TUG1 is expected to be a biomarker and therapeutic target for the diagnosis and prognosis of liver cancer.

MicroRNAs play an important role in the occurrence and pathological processes of various tumors. As a member of the miRNA family, miR-29a plays an important role in HCC. Our previous studies have shown that miR-29a can inhibit the biological functions of liver cancer cells, such as metastasis, invasion, and proliferation 15, 17, 20. Additionally, its expression has a close relationship with tumor TNM staging, multifocal tumors, and venous invasion. After downregulating miR-29a in HCC cells, we found that the translocation, invasion, and migration of HCC-LM3 and MHCC-97H cells increased, their proliferative ability was enhanced, and their apoptosis ability weakened. This is consistent with our previously reported results. Moreover, it is verified that miR-29a acts as a tumor suppressor gene to control the occurrence and development of tumors^34^, ^35^. We found that TUG1 was negatively correlated with the expression of miR-29a by data alignment. Moreover, we found that TUG1 was highly expressed in our liver cancer samples using qRT-PCR, whereas miR-29a was lowly expressed in HCC. The higher the TUG1 expression is, the lower the expression of miR-29a would be. We used immunofluorescence and dual luciferase assay to show that miR-29a is a downstream target gene of TUG1. Recently, TUG1 has been found to act as a microRNA (miRNA) sponge to indirectly regulate the expression of miRNA target genes and plays a leading role in the progression of various cancers. However, it is unclear how the combination of TUG1 and miRNA affects the progression of liver cancer. The primary objective of this experiment was to study the involvement of TUG1 and miR-29a in the progression of HCC.

In the Asian population, IFITM3 rs12252 is associated with the severity of influenza infection. However, recently, there has been growing evidence that IFITM3 is closely related to the development and prognosis of several tumors 28, 30. For example, Li et al. showed that the expression of IFITM3 was significantly upregulated in colon cancer with a crucial relationship with its development and metastasis 27. Andreu et al. have shown that IFITM3 is significantly overexpressed in the rectum and can rapidly activate the β-catenin signaling pathway^36^. In esophageal squamous cell carcinoma, patients with high expression of IFITM3 are more likely to develop lymph node metastasis after surgery. Downregulation of the expression of IFITM3 inhibited the growth of breast cancer cells and colony formation. In liver cancer, IFITM3 can promote tumor metastasis, and patients with high expression of IFITM3 have a relatively poor prognosis. Therefore, IFITM3 has been shown to play a key role in tumorigenesis. In our study, it was found that the expression of IFITM3 in HCC was significantly increased, and its expression was related to the prognosis of HCC, and it was also closely related to TNM staging and tumor venous infiltration. We also confirmed through functional experiments that the expression of IFITM3 has a significant relationship with the invasion, metastasis, proliferation, and apoptosis of HCC cells. We used bioinformatics analysis and related website predictions, using dual-luciferase reporting and other technologies to carefully verify that miR-29a can inhibit the expression of IFITM3, and IFITM3 is the downstream target gene of miR-29a. Therefore, we first proposed that TUG1 affects the expression of IFITM3 by regulating miR-29a, which in turn affects the occurrence and development of HCC.

TUG1, miR-29a, and IFITM3 are closely related to the occurrence and development of several tumors. However, it is unclear whether there a relationship exists between these three. The primary aim of the present study was to explore the impact of these three factors on liver cancer. First, using multiple databases, we found that TUG1, miR-29a, and IFITM3 are differentially expressed in HCC tissues and adjacent tissues. Furthermore, we found that the overexpression of TUG1 and IFITM3 could affect the prognosis of patients. Next, we verified the expression of these three factors in HCC tissues and cell lines. We found that the downregulation of TUG1 enhanced the expression of miR-29a and reduced the expression of IFITM3. Downregulation of TUG1 in HCC cells reduced cell invasion and metastasis, decreased proliferation, and increased apoptotic capacity, whereas the simultaneous downregulation of miR-29a enhanced cell invasion and metastasis, proliferation ability, and weakened cell apoptosis ability. Moreover, the expression of IFITM3 increased. Next, we performed a dual luciferase assay of TUG1 and miR-29a, and miR-29a and IFITM3, which confirmed that miR-29a is the downstream target gene of TUG1, and IFITM3 is the downstream target gene of miR-29a. It can be seen that IFITM3 is not only negatively regulated by miR-29a but also positively regulated by TUG1. This result indicates that TUG1 can regulate IFITM3 by competitively binding to miR-29a. Moreover, we have verified the above results through functional experiments. Based on these results, we conclude that TUG1 can be used as a ceRNA to competitively bind miR-29a to promote the expression of IFITM3, which in turn affects the invasion, metastasis, proliferation, and apoptosis of HCC. Finally, we confirmed our results in animals. Therefore, HCCs that overexpress TUC1 and IFITM3 and those that do not overexpress miR-29a could be more aggressive and malignant, and such patients are more prone to multifocal and intrahepatic recurrence.

To summarize, the results of the present study show that TUG1 can be used as a ceRNA to competitively bind miR-29a to regulate IFITM3, which in turn affects the occurrence and development of HCC. This study further elucidated the molecular mechanism of liver cancer and provided new therapeutic targets for the diagnosis and treatment of liver cancer.

## Conclusions

Based on the data provided in this study, TUG1 is a tumor-promoting gene of HCC. Downregulation of TUG1 can significantly inhibit the growth and metastasis of HCC cells, and the expression of TUG1 is closely related to the prognosis of HCC. In addition, TUG1 can be used as a ceRNA to competitively bind miR-29a to regulate the expression of IFITM3, thereby affecting the occurrence and development of HCC. In summary, TUG1 could be a potential therapeutic target for HCC, which can provide a new direction for the treatment of HCC.

## Supplementary Material

Supplementary figures and tables.

## Figures and Tables

**Table 1:** Clinicopathological characteristics of HCC patients and the expression of TUG1

Clinicopathologic characteristics	n	Overexpression (n=41)	Non-overexpression (n=24)	p-value
**Age (years)**				0.714
≤51	29	19	10	
>51	36	22	14	
**Sex**				0.225
Male	39	21	16	
Female	26	20	8	
**Tumor size**				**0.004**
≤5 cm	21	8	13	
>5 cm	44	33	11	
**TNM stage**				**0.011**
I-II	30	14	16	
III-IV	35	27	8	
**Tumor multifocal**				0.613
Absent	27	18	9	
Present	38	23	15	
**Venous invasion**				**0.026**
Absent	29	14	15	
Present	36	27	9	
**HBsAg**				0.685
Negative	25	15	10	
Positive	40	26	14	
**AFP (ng/ml)**				0.067
≤400	31	16	15	
>400	34	25	9	
**Cirrhosis**				0.217
Absent	21	11	10	
Present	44	30	14	

**Table 2:** Clinicopathological characteristics of HCC patients and the expression of IFITM3

Clinicopathologic characteristics	n	Overexpression (n=41)	Non-overexpression (n=24)	p-value
**Age (years)**				0.377
≤51	29	20	9	
>51	36	21	15	
**Sex**				0.059
Male	39	21	18	
Female	26	20	6	
**Tumor size**				0.074
≤5 cm	21	10	11	
>5 cm	44	31	13	
**TNM stage**				**0.002**
I-II	30	13	17	
III-IV	35	28	7	
**Tumor multifocal**				0.590
Absent	27	16	11	
Present	38	25	13	
**Venous invasion**				**0.003**
Absent	29	14	15	
Present	36	27	9	
**HBsAg**				0.685
Negative	25	15	10	
Positive	40	26	14	
**AFP (ng/ml)**				0.776
≤400	31	19	12	
>400	34	22	12	
**Cirrhosis**				0.678
Absent	21	14	7	
Present	44	27	17	

## Abbreviations

TUG1: Taurine upregulated gene 1
miR-29a: microRNA 29a
IFITM3: interferon-inducible transmembrane protein 3
HCC: hepatocellular carcinoma
qRT-PCR: quantitative real-time polymerase chain reaction
DR: dual-luciferase reporter
FISH: fluorescence *in situ* hybridization
