# TUG1 promotes the expression of IFITM3 in hepatocellular carcinoma by competitively binding to miR-29a: Erratum

**DOI:** 10.7150/jca.99135

**Published:** 2024-08-08

**Authors:** Weiwei Liu, Qian Feng, Wenjun Liao, Enliang Li, Linquan Wu

**Affiliations:** Department of Hepatobiliary Surgery, the Second Affiliated Hospital of Nanchang University, 1 Mindle Road, Nanchang Jiangxi 330006, P.R. China.

In our published paper, due to the error in editing images, Figure 6D,7F,9E appeared to have incorrect use of pictures, and we provide the updated pictures below. At the same time, since the Figure legend in Figure 5 is not accurate enough, we also correct it. We deeply regret and sincerely apologize for these errors and any inconvenience that may have been caused to the readers and editors of this journal.

**Figure 5 correct legend**: ** The relationship between miR-29a and IFITM3 A.** Immunofluorescence analysis of IFITM3 expression in HCC-LM3 and HL-7702 at a scale of 200 mm. B. qRT-PCR was used to detect the expression of miR-29a and IFITM3 in HCC. C. The prognosis of patients with high expression of IFITM3 is worse than that of patients with low expression of IFITM3. D. Changes in IFITM3 mRNA levels after miR-29a inhibitor and IFITM3 siRNA transfection. E. Changes in IFITM3 protein levels after miR-29a inhibitor and IFITM3 siRNA transfection. F. The Targetscan website is used to predict the relationship between miR-29a and IFITM3.

## Figures and Tables

**Figure A:**
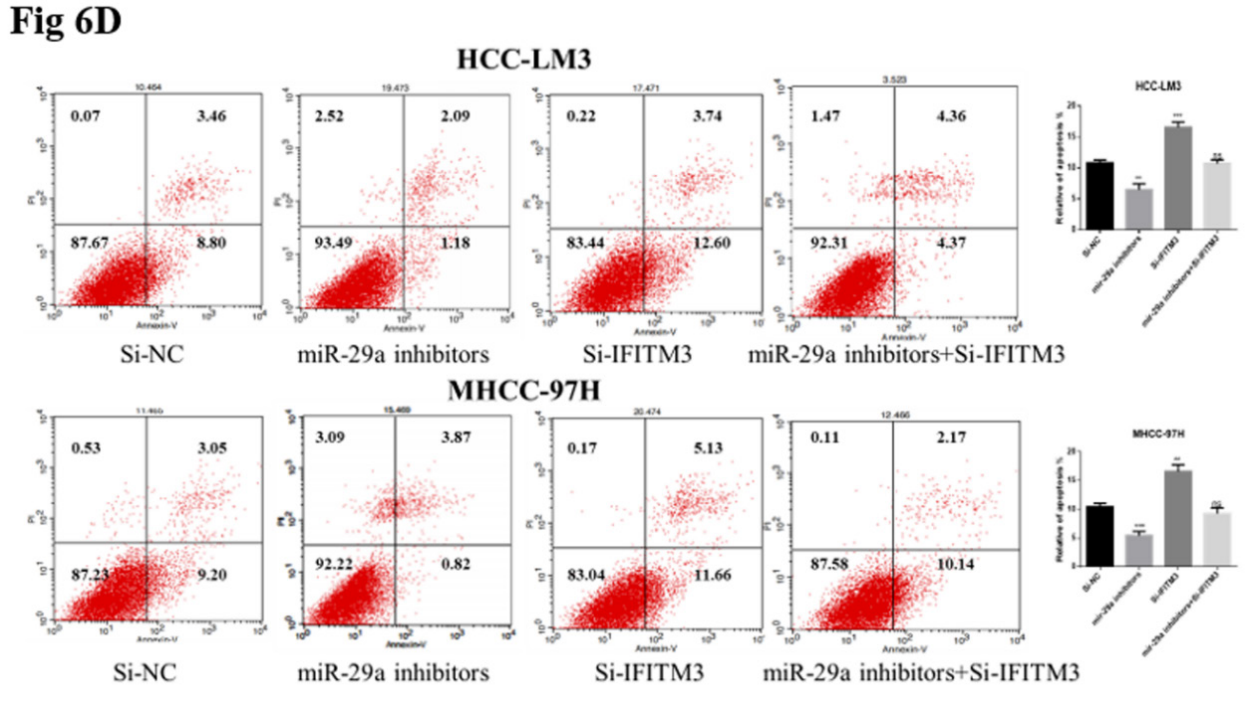

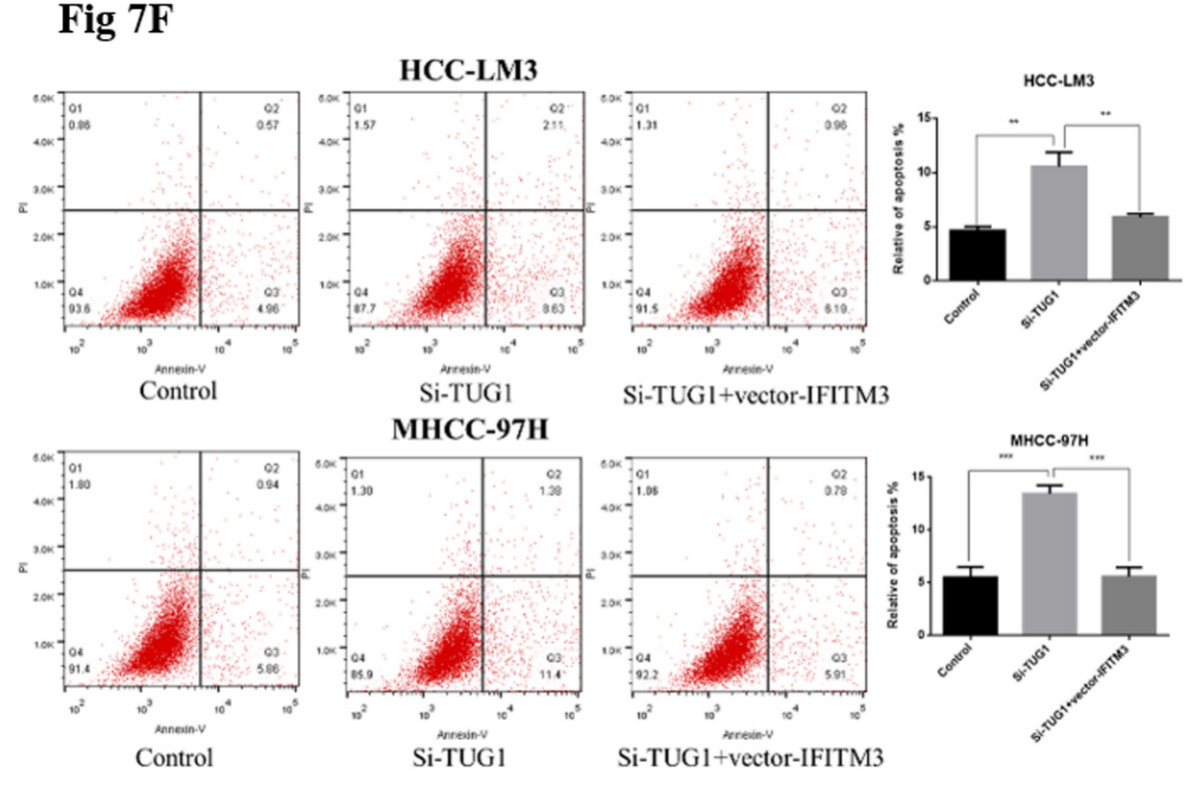

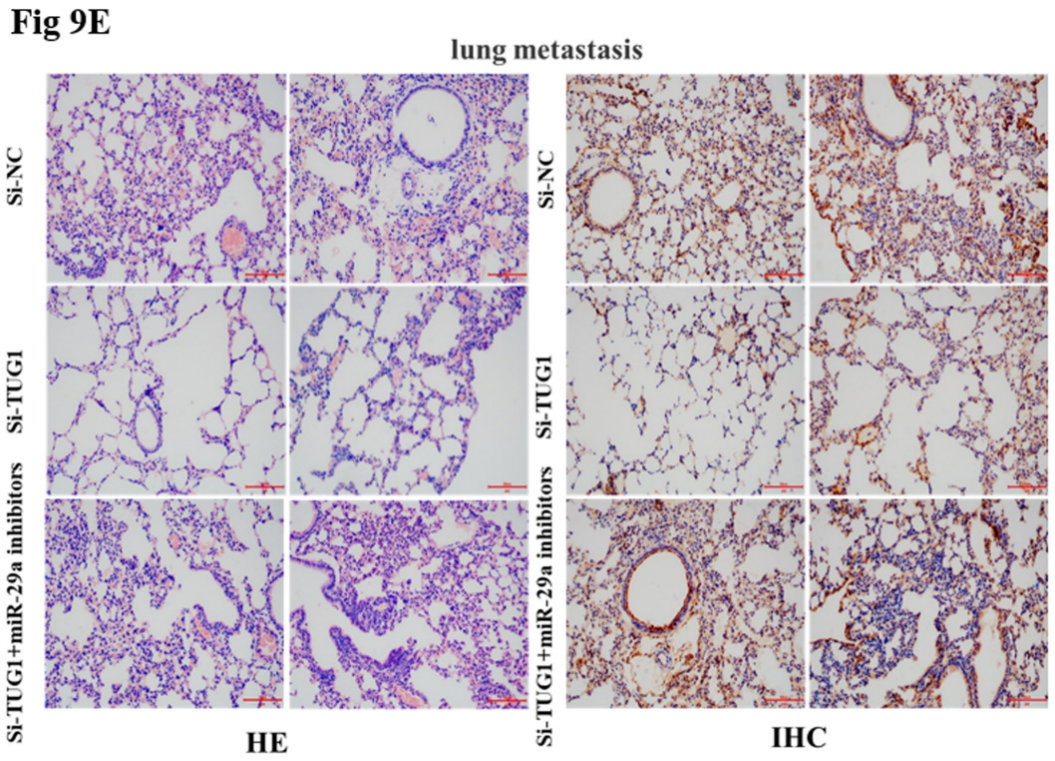
Correct images for figures 6D,7F,9E. **6D.** changes in apoptosis rate of cells transfected with miR-29a inhibitor and IFITM3 siRNA.** 7F.** changes in apoptosis rate of cells transfected with si-TUG1 and IFITM3 vectors. **9E.** Lung metastasis-related detection. H&E staining of lung tissue for histological analysis.

