# Supplementary material for: TUG1 promotes the expression of IFITM3 in hepatocellular carcinoma by competitively binding to miR-29a
Source: J Cancer. 2021 Oct 2;12(22):6905–20. doi: 10.7150/jca.57477 (PMC8517998; doi:10.7150/jca.57477)
Supplement: Supplementary file 1 — Supplementary figures and tables. [file jcav12p6905s1.pdf]

Supplementary Figure 1. A. The relationship between the expression of TUG1 and TNM staging. B.The expression of TUG1 and mir-29a obtained from the database. C. The expression of IFITM3 in HCC downloaded from the GEPIA2 database. D. The expression of IFITM3 in HCC. E. Based on the results of qRT-PCR, the relationship between the expression of TUG1 and IFITM3 is shown.

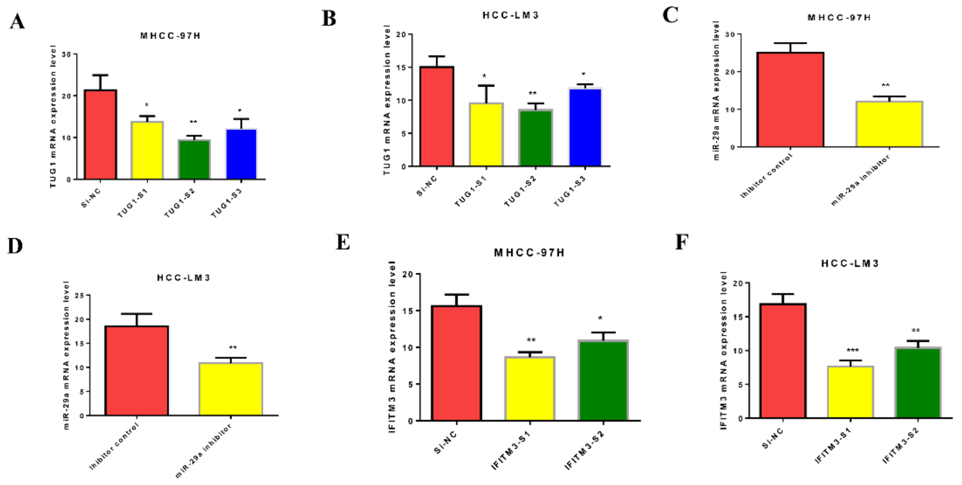


Supplementary Figure 2. Before performing functional experiments, first transfect all interfering fragments into HCC cells, verify the effect of SiRNA by qRT-PCR, and select the best one. A.B: TUG1 3 SiRNA selection. C, D: Test the inhibitory effect of miR-29a inhibitors in two cell lines. E, F: IFITM3 3 SiRNA selection. *$p < 0.05$, **$p < 0.01$, ***$p < 0.001$, ****$p < 0.0001$.

The p-value represents the $\chi^2$ test performed to show the difference between the expression of TUG1 and IFITM3 in each group. HCC, hepatocellular carcinoma; AFP, α-fetoprotein; HBsAg, hepatitis B surface antigen; TNM, tumor–node metastasis; n, number of cases.

Table S1. Selection of downstream target genes of TUG1.

Table S2. List of primer sequences.

**Selection of downstream target genes of TUG1**

| Gene | miRNA | Pr. score |
|---|---|---|
| TUG1 | hsa-miR-320c | 0.815 |
| TUG1 | hsa-miR-29b-1-5p | 0.634 |
| TUG1 | hsa-miR-5006-3p | 0.611 |
| TUG1 | hsa-miR-135a-5p | 0.729 |
| TUG1 | hsa-miR-18a-5p | 0.692 |
| TUG1 | hsa-let-7d-5p | 0.718 |
| TUG1 | hsa-miR-9-3p | 0.810 |
| TUG1 | hsa-miR-27a-3p | 0.815 |
| TUG1 | hsa-miR-224-5p | 0.688 |
| TUG1 | hsa-miR-1305 | 0.937 |
| TUG1 | hsa-miR-21-5p | 0.680 |
| TUG1 | hsa-miR-548ay-5p | 0.863 |
| TUG1 | hsa-miR-588 | 0.703 |
| TUG1 | hsa-miR-16-1-3p | 0.625 |
| TUG1 | hsa-miR-29a-3p | 0.941 |
| TUG1 | hsa-miR-186-5p | 0.764 |

**Primer sequence**

| Target Gene | Primer(5’-3’) |
|---|---|
| TUG1 | F:5’- GCATCTTTGCCCACATACACC -3’<br>R: 5’- GCCTGCTCTCTAAACTCCTCCTT -3’ |
| miR-29a | F: 5’-TGCGCTAGCACCATCTGAAAT-3’<br>R: 5’-CAGTGCAGGGTCCGAGGT-3’ |
| IFITM3 | F:5’ - ACTGTCCAAACCTTCTTCTCTCC -3’<br>R: 5’-TCGCCAACCATCTTCCTGTC-3’ |
| GAPDH | F: : 5’-TATGATGATATCAAGAGGGTAGT-3’<br>R: 5’-TGTATCCAAACTCATTGTCATAC-3 |
| U6 | F: 5’ -CTCGCTTCGGCAGCACA-3’<br>R:5’ -AACGCTTCACGAATTTGCGT-3’ |
